# Conversion Rate Prediction Based on Text Readability Analysis of Landing Pages

**DOI:** 10.3390/e23111388

**Published:** 2021-10-23

**Authors:** Ruslan Korniichuk, Mariusz Boryczka

**Affiliations:** Institute of Computer Science, Faculty of Science and Technology, University of Silesia in Katowice, Będzińska 39, 41-200 Sosnowiec, Poland; mariusz.boryczka@us.edu.pl

**Keywords:** classification, conversion rate prediction, landing pages, machine learning, marketing communications, readability indices

## Abstract

Digital marketing has been extensively researched and developed remarkably rapidly over the last decade. Within this field, hundreds of scientific publications and patents have been produced, but the accuracy of prediction technologies leaves much to be desired. Conversion prediction remains a problem for most marketing professionals. In this article, the authors, using a dataset containing landing pages content and their conversions, show that a detailed analysis of text readability is capable of predicting conversion rates. They identify specific features that directly affect conversion and show how marketing professionals can use the results of this work. In their experiments, the authors show that the applied machine learning approach can predict landing page conversion. They built five machine learning models. The accuracy of the built machine learning model using the SVM algorithm is promising for its implementation. Additionally, the interpretation of the results of this model was conducted using the SHAP package. Approximately 60% of purchases are made by nonmembers, and this paper may be suitable for the cold-start problem.

## 1. Introduction

Regardless of whether we run a small business or a large corporation, we probably engage in marketing communications. Marketing communications include advertising, promotions, sales, branding, campaigning, and online promotion.

The most important thing about advertising is the text [1]. Readability, in turn, makes some texts easier to read and understand than others. Readability is often confused with legibility, which concerns typeface and layout, and which refers to the visual clarity of individual symbols.

Mc Laughlin (creator of the SMOG readability index) defined readability as “the degree to which a given class of people find certain reading matter compelling and comprehensible” [2].

Dale and Chall’s [3] definition may be the most exhaustive: “the sum total (including the interactions) of all those elements within a given piece of printed material that affects the success that a group of readers have with it; the success is the extent to which they understand it, read it at an optimal speed, and find it interesting”.

It is worth noting that the term “readability” used in this work is identified with the concept of “comprehensibility”, which is, however, a kind of simplification. The issue of text comprehensibility is broad and multidomain, as education, social conditions, the context of the message, and imagination are important for text comprehensibility.

The notion of a “landing page” also requires clarification. In digital marketing, a landing page is a standalone web page created for the purposes of a marketing or advertising campaign. It is the place where the user “lands” after clicking on a link in an e-mail or an ad from Google, YouTube, Facebook, Instagram, or similar places on the web.

By landing page conversion, we mean the action that is recorded when someone interacts with the text (e.g., reads a product description) and then performs an action that is valuable to the company, such as completing a purchase or calling its office.

The aim of this paper was to investigate whether it is possible to predict the conversion rate (CVR) by analyzing the landing page text for its readability.

Approximately 60% of purchases are made by nonmembers [4]. That is why this article, among other things, is devoted to the cold-start problem, where the purchasing behavior prediction is complicated by the lack of abundant information for first-time visitors.

It should be noted here that the topic of this paper is not about conversion rate optimization (CRO), including the CRO of landing pages. The purpose was also not to investigate a wide set of variables that may determine the conversion rate (e.g., writer invariant, sentiment, elements of a landing page design, domain name, and product price). We were interested in studying the impact of text readability on the conversion rate, and conversion rate prediction.

The data were obtained from Landingi Sp. z o.o. (https://landingi.com, accessed on 22 October 2021) with the support of Błażej Abel—CEO and founder of the company. Landingi is a platform for everyday marketing activities with landing pages. The data concern the content of landing pages and their conversion rates. For conversion prediction, machine learning algorithms were used. We built five machine learning models. The accuracy of the presented machine learning model using the SVM algorithm is promising for its implementation. Moreover, the SHAP package was used to interpret the results of this model.

This paper found that a detailed analysis of text readability is capable of predicting conversion rates. There are readability indices with positive and negative correlation to conversion. Additionally, a single readability index can have both a positive and a negative impact on conversion.

The remainder of the paper is organized as follows: Section 2 provides a brief description of the problem behind the paper and a reference to other similar studies; Section 3 presents a description of the input data for the experiments, a description of the selected methods for assessing text readability, and the algorithms used to build and interpret the machine learning model; Section 4 describes the research conducted, the results obtained, and their interpretation; the paper concludes with a summary, included in Section 5.

## 2. Problem Description and Related Works

Hopkins (one of the great advertising pioneers) believed that a scientific approach to advertising should be applied. Hopkins [5] said, “The time has come when advertising has in some hands reached the status of a science. It is based on fixed principles and is reasonably exact. The causes and effects have been analyzed until they are well understood”.

The text in an online store, on a landing page, in an e-mail, or in an advertisement must have the effect of increasing sales. According to a study by Hughes (an authority in the area of modern marketing), traditional advertising reaches only 0.06% of the target population. Ninety-six percent of all visits to a website do not end with product purchases [6].

Conversion rates average approximately 2–4% across online retail sites [4,6,7]. Given the dramatic growth in online usage coupled with the historically low conversion rates, any increase in conversion rate could greatly affect a firm’s profitability [6,7]. Deceptively small increases of even 1% in conversion rate at retailers such as Amazon (www.amazon.com, accessed on 22 October 2021) can translate into millions of dollars in sales revenues [4,7].

Estimating CVR accurately not only helps increase profits but also improves the experience of audiences on the website so that it attracts more users to visit the website [8,9]. Therefore, predicting the CVR accurately has become a core task of advertising [8,9,10].

For this purpose, a number of techniques are used, including focus group interviews and A/B testing. However, an increasing number of experts believe that group interviewing and related techniques have become ineffective [11].

Atalay, El Kihal, and Ellsaesser [12] in their research showed that the syntactic complexity of the message used affects its persuasiveness. In one experiment, participants were randomly assigned to read one of two texts persuading them to wear a helmet while cycling. One text had low syntactic complexity, while the other had high syntactic complexity. The number of participants who changed their attitude toward wearing a helmet after reading the text was measured.

The relative change in attitudes of the group reading the text with high syntactic complexity was significantly lower than the relative change in attitudes of the group reading the text with low syntactic complexity. This difference in individual attitude change was significant and constitutes evidence that high syntactic complexity is harmful toward the persuasiveness of the message.

Another experiment showed that lowering the syntactic complexity of a Facebook advertisement increased the click-through rate (CTR). It was found that CTR was significantly higher for a version of the same advertisement with lower syntactic complexity (1.07%) than the CTR of the original advertisement created by the agency (0.76%).

According to a report by Unbounce “The Unbounce Conversion Benchmark Report 2020” [13], easier-to-read landing pages had higher conversion rates than pages that were more difficult to read. However, easy-to-read can vary widely from industry to industry. There are also exceptions to this rule, which were listed in the report.

There are currently no more published research results on predicting conversion rate on the basis of text readability. In most cases, the techniques used for CVR and CTR prediction share some commonalities; however, the conversion behavior is more complex than click behavior [8]. That is why studies on CTR prediction [14,15,16,17] are not suitable for CVR prediction. Increasingly, marketing professionals are ultimately interested in responses subsequent to the click, such as purchase [18].

For example, Du et al. [19] and Xia et al. [9] studied the click-through rate (CTR) prediction using certain groups of variables:

- User: describes user behavior and personal information.
- Context: includes ad click-based features: click time, purchase time, search result page number, and predicted category.
- Advertising product: describes the product information of the ad: category, brand, price, collected count, sales count, and displayed count.
- Shop: describes the information of the shop which sells the product: review numbers, rate of positive reviews, star level, score of service, and score of delivery.

This approach can be considered complicated due to the wide set of data that must be collected and the corresponding cold-start problem [20], i.e., the lack of abundant information for first-time visitors.

Gong et al. [21] in their research used time-series analysis to predict the conversion rate on an online shopping website that provides consumer-to-consumer (C2C) retail. One of the advantages of this approach is the ease of implementing this type of solution for companies. However, time-series analysis needs context, for example, data such as click time and product purchase time, which is not important from the point of view of the article presented.

Agarwal et al. [22] estimated CVR by utilizing the dependency between audience information and ad–publisher pair [8]. Rosales et al. [23] provided a detailed analysis of conversion rates in the context of nonguaranteed delivery targeted advertising [8]. Ludwig et al. [7] studied how the affective content and the linguistic style of product reviews influence the conversion rate [4]. Haans et al. [24] examined the impact of evidence type on conversion rate in a search engine advertising setting and found that causal evidence results in higher conversion rates than other types of evidence.

Chapelle [25] observed that the time delay between impression and click is so short, while the time delay between impression and conversion is so much longer, maybe days or weeks. Therefore, they proposed a CVR prediction model by utilizing the time delay [8]. Cezar et al. [26] examined the impact of review rating (location rating and service rating), recommendation, and search listings on converting browsers into customers. Jiang et al. [8] included the potential impact of creative in helping the CVR prediction task and found that some creatives lead to ads gaining high CVR, but some creatives are the opposite.

A rich set of variables that may determine the purchasing behaviors have been defined, evaluated, and used as the feed of prediction models, including the demographics of the customer, historical browsing, detailed browsing behavior, purchasing behavior, repeat visitation, social contacts and friends, and virtual community [4].

IT companies also invest in commercial solutions for predicting the conversion rate. For example, Baidu (www.baidu.com, accessed on 22 October 2021)—one of the largest artificial intelligence and internet companies in the world—has deployed a powerful reinforcement learning-based infrastructure that can increase click-through rates and conversions [27]. However, there are currently no published research findings on the use of reinforcement learning to predict conversion rate.

Landing pages were selected as the object for the experiments due to the fact that the length of the text (minimum 100 words) contained on landing pages allows for a credible analysis of its readability compared to, for example, the length of an SMS text (around 20 words [28]).

## 3. Data and Methodology

The assumption was that the texts on the landing pages from the dataset obtained from Landingi are written in English. Unfortunately, we were not able to obtain data from a single country, for example, the United States; however, only English-language texts were subjected the final analysis.

In our experiments, we aimed to investigate whether it is possible to predict the conversion rate (CVR) of a landing page by analyzing its text for readability. The next steps concern data preparation, data verification, readability indices, machine learning algorithms used in experiments, and interpretation of the results of machine learning models.

### 3.1. Data Preparation

**Figure 1:**
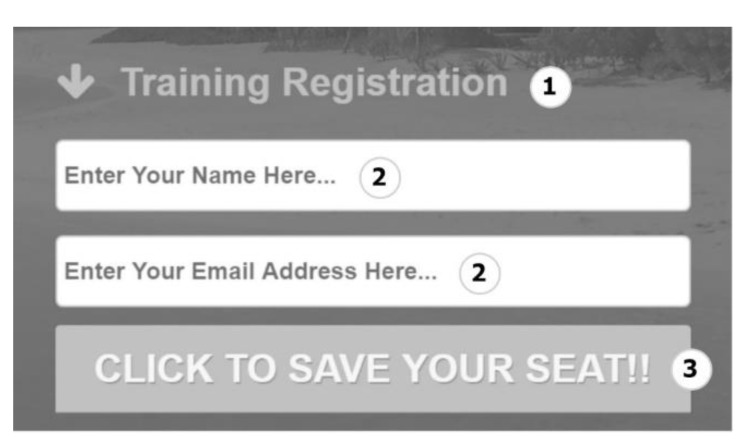
Visualization of the fields with text.

The most important task in data preparation was to export text data from the landing page content saved in JSON format. First, the parser extracted text from the *text*, *placeholder*, and *input/button* fields, saving it into separate blocks. The *text* represents the content of the landing page text field, *placeholder* is the text of a short hint describing the expected value of the input field, and *input*/*button* represents the text placed on the landing page button. Figure 1 contains a visualization, where: 1 marks the *text* field, 2 marks the *placeholder* fields, and 3 marks the *input*/*button* field.

As part of the second step, we applied filtering to the text data. We removed text blocks containing fewer than two words. Most often, these were blocks from buttons and placeholders. The motivation behind this step was that blocks containing fewer than two words would have a very high impact on readability indices in further analysis. For example, the average number of words per sentence would significantly decrease.

We used an in-house regular expression to extract single words of English. We subsequently combined the individual text blocks for each landing page into a whole for further detailed analysis.

### 3.2. Data Verification

One of the data verification steps was to check the dominant language for each landing page. The acquired data were marked as containing English (“en”), Polish (“pl”), Russian (“ru”), and Portuguese (“pt”). We should recall here that only texts in the English language were subjected to the final analysis.

Amazon Comprehend (https://aws.amazon.com/comprehend, accessed on 22 October 2021) language detection was used to check the dominant language. The service uses a pretrained machine learning model to examine and analyze a document to gather insights. This model is continuously trained on a large body of the text so that there is no need to provide training data.

### 3.3. Readability Indices

A readability index is a measure related to the difficulty of text perception by the reader. A readability index can be calculated on the basis of various parameters: sentence/word length, number of polysyllabic/multicharacter/difficult words, etc.

The readability indices used in this study can be divided into several groups. The first group is based on sentence length counted in words and word length counted in syllables. Longer sentences and longer words are more complex for reading and comprehension. The first group includes the Flesch [29], Flesch–Kincaid [2], Fog [30], SMOG [31], and Strain [32] readability indices.

The second group is based on sentence length counted in words and word length counted in characters. These include the Automated Readability Index (ARI) [33], Coleman–Liau [34], Lix [35], and Rix [35] readability indices.

The next readability index used in this paper, the New Dale–Chall [36,37], is unique. This index is based on the sentence length counted in words and the number of difficult words. Initially, this readability index was based on a list of 763 words that every statistical American student is required to understand before their senior year. Words that are not on this list are considered difficult to understand. Over time, the index improved and, by 1995, the word list was expanded to 3000 words.

Another readability index, the Bormuth [2], combines the approaches of the New Dale–Chall readability index and the second group indices.

It should be noted here that the aim of this paper was to investigate whether it is possible to predict the conversion rate by analyzing the landing page text for its readability, rather than a comprehensive review of and detailed instructions on how to build readability indices.

The lengths of sentences and words are convenient and credible indicators of readability and fit neatly into a formula but are not comprehensive measures. The various factors that make a text coherent are difficult to quantify. Remember that readability formulas cannot establish the depth of the ideas inherent in a text [38].

Measures of word and sentence length are sometimes not the most accurate indicators of difficulty. White [39] in his essay “Calculating Machine”, recounted his reaction when he received a “reading-ease calculator” developed by General Motors based on the Flesch readability index. “Communication by the written word”, writes White [39], “is a subtler (and more beautiful) thing than Dr. Flesch and General Motors imagine”.

### 3.4. Machine Learning Algorithms Used in Experiments

The naïve Bayes classifier [40] is a simple probabilistic classifier based on the application of Bayes’ theorem with the assumption of mutual independence of features. An implementation of the Gaussian naïve Bayes algorithm was used for classification.

Support vector machine (SVM) [41] is a method the basis of which is the concept of a decision space that is partitioned by creating boundaries that separate objects that belong to different classes. In the experiments, a nonlinear SVM classifier with radial basis function kernel (RBF) was used. The applied implementation was based on the machine learning library LIBSVM [42].

Random decision forests [43] are a machine learning method that involve constructing multiple decision trees during learning time and generating a class that is the dominant class of each tree.

Logistic regression is used in statistics, among others, when a variable takes only two values. In this paper, the logistic regression with regularization was used. The regularization is based on the limited memory BFGS (L-BFGS) method [44].

The *k*-nearest neighbors (*k*-NN) algorithm [45] is a neighbor-based classification—a type of instance-based learning or nongeneralized learning. It does not attempt to construct a general internal model but simply stores instances of the training data. Classification is computed from a simple majority vote of the nearest neighbors of each point.

We should recall here that the purpose of this article was to show that the machine learning approach can predict landing page conversion by analyzing its text for readability, rather than detailed instructions on how to build a state-of-the-art machine learning model for conversion rate prediction.

### 3.5. Interpretation of Machine Learning Models

SHAP (Shapley Additive Explanations) is a method to explain individual predictions [46,47]. SHAP assigns each feature an importance value for a particular prediction. SHAP is based on the theoretically optimal Shapley values. The Shapley value is a solution concept in cooperative game theory. To each cooperative game, it assigns a unique distribution of a total surplus generated by the coalition of all players.

### 3.6. Remark Concerning COVID-19

The collected data concern landing page content and conversions dating from before the spread of COVID-19 and its economic impact. The possibility that, in some industries, conversion rates may be slightly different now is assumed, e.g., air transport, travel, catering and restaurants, events and meetings, and fitness.

## 4. Experiments

On the basis of existing business problems, we first decided in our study to analyze the readability of landing page texts using, among other things, existing readability indices for the English language.

Secondly, we decided to analyze whether there was a correlation between the conversion rate and the readability of landing pages. If such a relationship existed, we wanted to see if we could predict the conversion rate by analyzing the readability of a landing page text. For conversion prediction, we used machine learning algorithms.

An important aim of our experiments was also to explore the possibility of relating to existing similar studies. In order to do so, we had to compare the source data to industry indicators and check whether it is true that the easier-to-read landing pages had higher conversion rates than the more difficult pages (according to the Unbounce report [13]).

The scheme of experiments was as follows: we started with data preparation. We compared our input data with industry indicators provided by Unbounce. Then, we calculated and analyzed the readability indices. In the next stage, we defined additional features for each landing page text and determined the relationships between conversion rate and features. Before the stage of building machine learning models, we solved the problem of strongly correlated features.

Among the various tasks of machine learning, we chose the classification problem. Classification makes the prediction of values possible as categories: yes/no, positive/negative, “conversion rate above the median”/“equal to or below the median”. In the next step, we calculated and compared the accuracy of different machine learning models. After building the models, we proceeded to the SVM model interpretation step using the SHAP package. As a result, we were able to relate the results to those published in similar works.

### 4.1. Data Cleaning

The initial dataset contained 2000 randomly selected examples. The conversion data concerning these examples were exported to a CSV file, as in Table 1, where *Index* denotes the index of the example, *Hash* denotes the unique identifier of the landing page, *Views* is the number of views for that landing page, *Conversions* is the number of conversions, and *CVR* is the conversion rate.

**Figure 2:**
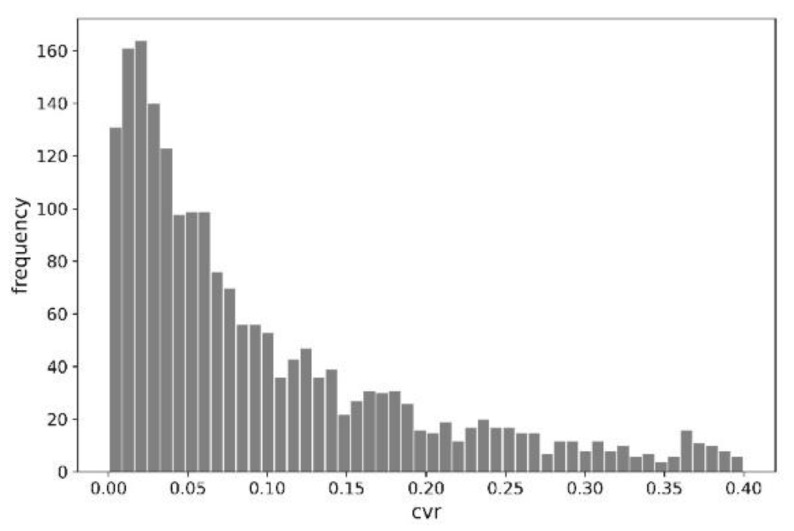
Distribution of conversion rates (2000 examples).

The random nature of the data was indirectly confirmed by the example indices in Table 1 and the distribution of conversion rates for 2000 examples, shown in Figure 2.

It is worth noting that the presented histogram is not coherent with a normal distribution (or Gaussian distribution). The distribution of the conversion rate variable represents an exponential distribution.

The content of the landing pages was exported to separate files in JSON format. Unfortunately, only content for 1531 of the 2000 examples was available.

Out of 1531 examples, 1030 examples were marked as containing English language. The others represented the “pl”, “ru”, and “pt” languages. Within these 1030 examples, only 505 examples had English as the dominant language, according to Amazon Comprehend language detection. A total of 502 out of 505 examples had a level of confidence above 0.9. We did not take three examples with a low level of confidence for further analysis. The level indicates the confidence level (a number between 0.00 and 1.00) that Amazon Comprehend has that a particular language is the dominant language in the input text.

We also checked 501 examples marked as not containing English, i.e., as “pl”, “ru”, or “pt”. However, 12 of them represented English as the dominant language according to Amazon Comprehend language detection. Each of these 12 examples had a confidence level above 0.9.

Thus, as a whole, we obtained 514 (502 + 12) examples in English. Of these, 465 examples were for landing pages with texts containing a minimum of 100 words. This number of words comes from the requirements for determining the Flesch readability index [29], Fog readability index [30], Coleman–Liau readability index [34], Fry readability graph [48], and Raygor readability graph [49].

Another filter was the number of landing page views. We excluded pages with fewer than 500 views. The motivation for this was to be able to compare with the indicators of the conversion report published by Unbounce [13]. As a result, we obtained 243 examples during the machine learning model building stage. This is because 243 examples out of 465 had a minimum of 500 views each.

### 4.2. Comparison of Source Data with Industry Indicators

We compared our input data with the results in a report by Unbounce “The Unbounce Conversion Benchmark Report 2020” [13]. The arithmetic mean for all analyzed landing pages (minimum 500 views) was 7.99%. By comparison, the Unbounce report indicated an arithmetic mean value equal to 9.7%. The median conversion rate for all analyzed landing pages (minimum 500 views) was 4.16%. The Unbounce report indicated a median conversion rate of 3.2%.

The median conversion rate varies by industry. For example, according to Unbounce, the median conversion rate for the catering and restaurants industry is 15.6%, whereas, for the medical practitioners industry, it is 6% [13]. Therefore, having an uneven example distribution across industries, we can expect different values of the arithmetic mean. Therefore, the difference in arithmetic mean and median between our input data and the data in the Unbounce report is primarily due to the different number of examples selected for each industry.

### 4.3. Readability Index Analysis

For the dataset obtained during the previous step, we calculated 11 readability indices with their interpretation (subsequently referred to as variables) for all landing pages (minimum 100 words). We noticed a correlation between the conversion rate and some of these variables. Table 2 compares the Pearson, Spearman, and Kendall correlation coefficients between conversion rate and readability index or its interpretation. We included only the variables with the highest level of correlation in the table.

Table 2 indicates that there were readability indices with both positive and negative correlation with the conversion rate. We determined the level of correlation between conversion rate and the New Dale–Chall readability index (*new_dale_chall_score*), Strain readability index (*strain_score*), and New Dale–Chall readability index interpretation (*new_dale_chall_class*) to be low.

In the next step, we decided to analyze the relationship between the conversion rate and the different indicators on which the readability indices are based.

### 4.4. Analysis of Additional Features

We determined additional features for each landing page text (minimum 100 words). At first, these were the particular variables from the readability index formulas. We added the following features:

- *asl_flesch* is the average sentence length (according to the Flesch readability index),
- *asl_fog* is the average sentence length (according to the Fog readability index),
- *asw_flesch* is the average number of syllables per word (according to the Flesch readability index),
- *ppw_fog* is the percentage of polysyllabic words (according to the Fog readability index).

Secondly, the features directly related to text readability were average sentence/word length, percentage of polysyllabic words (≥3 syllables), percentage of multicharacter words (>6 characters), and percentage of difficult words (according to the New Dale–Chall readability index).

Additionally, we added features indirectly related to text readability:

- *pew* is the percentage of echomimetic (onomatopoeic) words,
- *psw* is the percentage of selling words,
- *puw* is the percentage of unique words.

Table 3 includes the full list of added features with a brief description and the Pearson correlation coefficient between the conversion rate and the feature.

**Figure 3:**
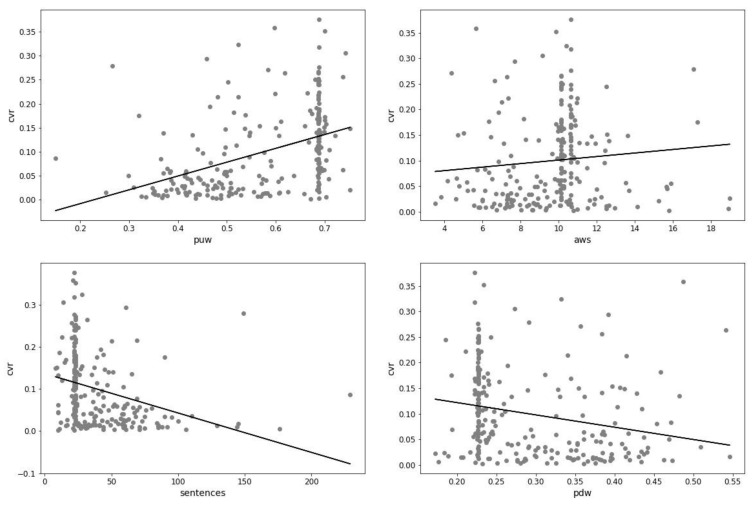
Linear regression with one variable (*puw*, *aws*, *sentences*, and *pdw* features) for conversion rate prediction.

Figure 3 contains a visualization based on linear regression. We chose this format to show the relationship between conversion rate and key features. The visualizations show that there is a positive correlation between the conversion rate and the features *puw* and *aws*. Moreover, there is a negative correlation between the conversion rate and the features *sentences* and *pdw*.

A low percentage of unique words can contribute to low conversion. Moreover, short sentences can cause low conversion. On the other hand, long text can also cause low conversion. Additionally, low conversion can be caused by a high percentage of difficult words (according to the New Dale–Chall readability index).

Furthermore, it is worth noting the strong correlation between the length of the text and the percentage of unique words present in it. A longer text contained a lower percentage of unique words (Pearson correlation coefficient: −0.739811).

We defined the level of correlation between the conversion rate and the percentage of unique words as medium. Since we had features with low and medium significance of correlation between conversion rate and feature, we decided to move on to building machine learning models.

### 4.5. Building Machine Learning Models

We first applied filtering to the list of features to solve the problem of strong correlation between them. Strongly correlated features generally do not improve the quality of machine learning models, but they affect specific models in different ways and to different degrees.

In the case of linear models (e.g., logistic regression), multicollinearity can provide solutions that are wildly varying and possibly numerically unstable. Random decision forests can be good at detecting interactions between different features, but highly correlated features can mask these interactions.

After applying filtering, the following 20 features were left: *ari_class*, *aws*, *bormuth_class*, *bormuth_score*, *coleman_liau_class*, *flesch_class*, *flesch_kincaid_class*, *fog_class*, *fog_score*, *lix_class*, *pew*, *pdw*, *ppw*, *psw*, *puw*, *rix_class*, *rix_score*, *sentences*, *smog_class*, and *strain_class*.

It should be noted here that the feature *readability score* was removed from the final list due to the fact that this feature is calculated as the arithmetic mean of all 11 readability indices, i.e., it does not enrich the data.

#### 4.5.1. Classification

We began preparing the machine learning models for the classification task by creating a *cvr_class* column to describe the classes. We assigned the data to class 1 or class 0 (true/false) according to the conversion rate value. Class 1 represents landing pages with a conversion rate above the median, while class 0 represents landing pages with a conversion rate equal to or below the median.

On the basis of the 20 features listed earlier and the conversion class *cvr_class*, we built machine learning models for classifying landing pages according to the predicted conversion.

Firstly, we built a model based on a naïve Bayes classifier. The accuracy of this model was 0.69863, which was promising for further analysis.

Secondly, we built models using the SVM, random decision forest, logistic regression, and *k*-NN algorithms. The SVM and *k*-NN algorithms achieved the highest accuracy. The accuracy for both models was 0.753425. After choosing an appropriate value of regularization parameter, the accuracy of the improved SVM model was 0.780822, and the *F*_1_ score for each class was 0.783784.

Table 4 shows the full list of built machine learning models along with their accuracy and *F*_1_ score for both classes.

#### 4.5.2. Interpretation of the SVM Model

To analyze the results, we used the KernelExplainer of the SHAP package. The mean absolute value of the SHAP values can show how much each feature contributed to the prediction of the value of the target variable.

**Figure 4:**
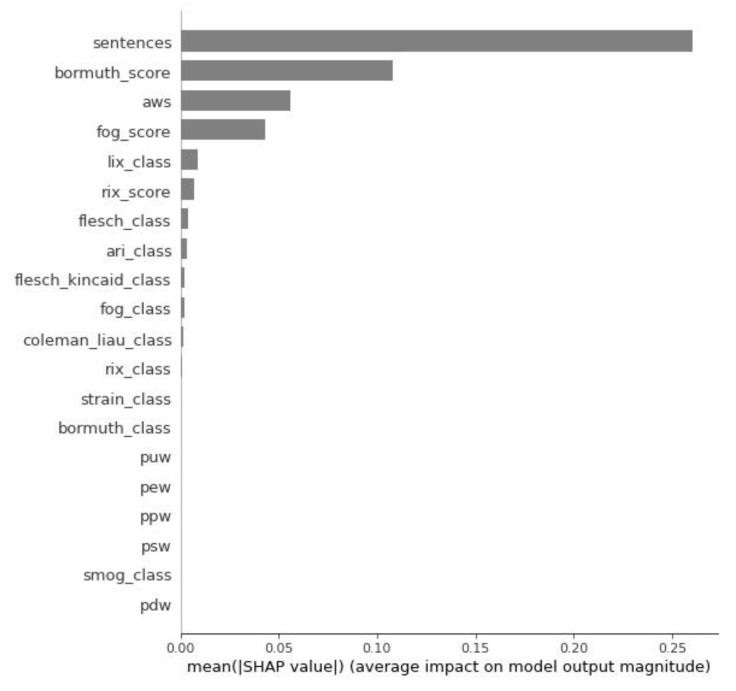
Global feature importance.

Figure 4 represents the feature importance graph. The graph lists the most significant features in descending order of importance. The top features contribute the most to the model. A lower feature is a weaker one, i.e., it has less predictive power. Thus, the four strongest features in our model were *sentences*, *bormuth_score*, *aws*, and *fog_score*.

**Figure 5:**
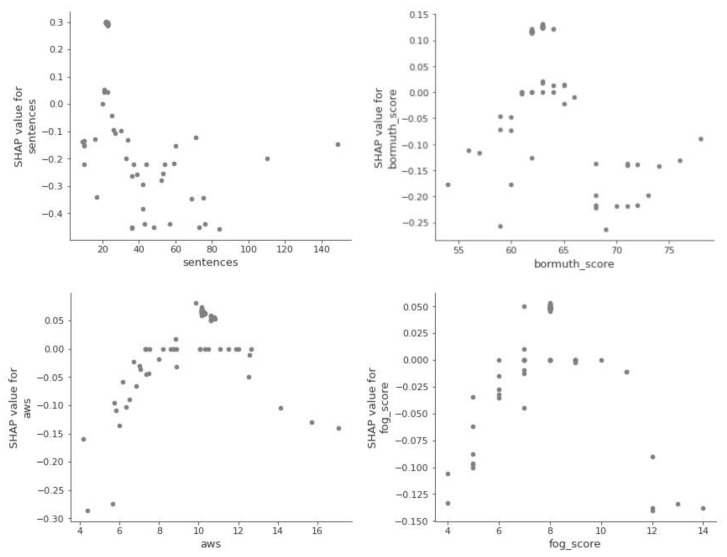
Dependence between features (*sentences*, *bormuth_score*, *aws*, and *fog_score*) and the target variable *cvr_class*.

Next, we look at the so-called dependency graphs, which show whether and what kind of relationship exists between the target and the object, i.e., whether it is linear or more complex. Figure 5 shows that the relationship between the aim and the object exists but is nonlinear.

**Figure 6:**
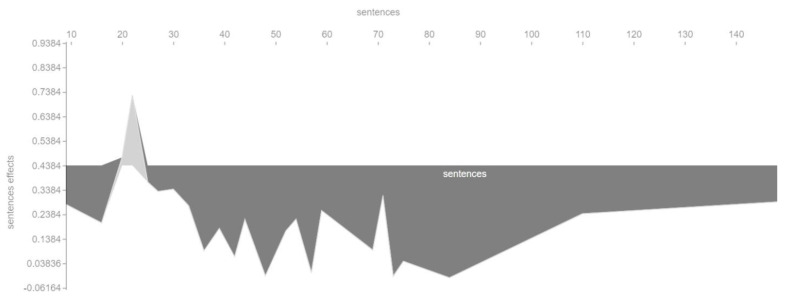
Positive and negative influence of the *sentences* feature on the target variable *cvr_class*.

**Figure 7:**
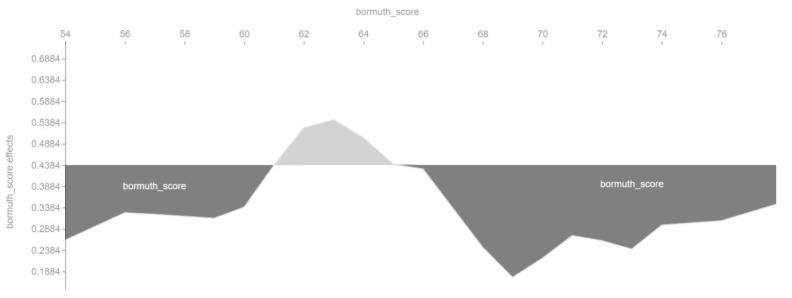
Positive and negative influence of the *bormuth_score* feature on the target variable *cvr_class*.

**Figure 8:**
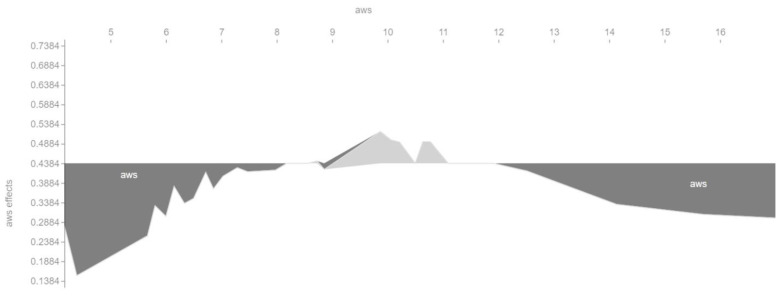
Positive and negative influence of the *aws* feature on the target variable *cvr_class*.

**Figure 9:**
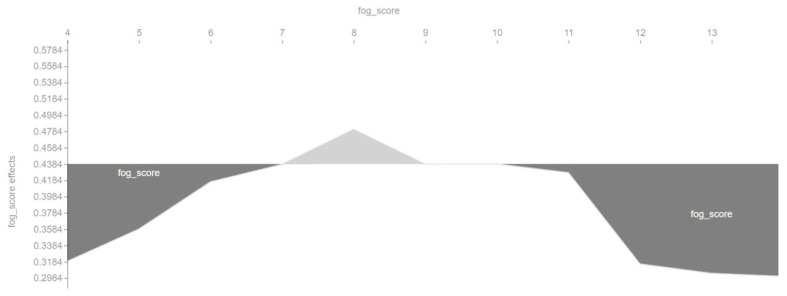
Positive and negative influence of the *fog_score* feature on the target variable *cvr_class*.

We also checked in a different way the influence of the main features on the output of the machine learning model. Figure 6, Figure 7, Figure 8 and Figure 9 show so-called force plots. On them, we can observe when the value of a feature has a positive or a negative effect on the value of the target variable *cvr_class*.

It is worth noting that this visualization also shows that the relationship between the conversion rate and the main features of the SVM model is nonlinear. For each feature, there are specific ranges of values in which the feature positively or negatively affects the value of the target variable *cvr_class*.

For example, Figure 6 shows that the length of a landing page can only positively affect the conversion rate within a certain range. The optimal value in this case is a landing page length of about 22 sentences. Both a too small and a too large number of sentences can in turn negatively affect the conversion rate.

Figure 7 shows that the optimal value of the Bormuth readability index is around 62–64. According to the interpretation of this index, this denotes a standard language because it is within the meaning range 58–71 of the Bormuth readability index interpretation table.

In turn, Figure 8 shows that the conversion rate is positively affected by an average sentence length of about 10–11 words, which is well below the standard sentence length of 17 words according to Watson Solomon [32] (independent communication consultant). Both very short and very long sentences can, therefore, negatively affect the conversion rate.

Figure 9, similarly to Figure 7, shows that the optimal value of the Fog readability index is around 8. According to the interpretation of this index, this denotes a simple language, already understandable for middle-school students. This is because this value is within the meaning range of 7–9 of the Fog readability index interpretation table.

### 4.6. Discussion of the Results

Summarizing the conducted research, the following can be concluded:

- The conversion rate values have an exponential distribution.
- The conversion rate of the input data used in the experiment (arithmetic mean: 7.99%; median: 4.16%) is comparable to the industry indicators from the Unbounce report (arithmetic mean: 9.7%; median: 3.2%) [13].
- There are readability indices with both positive and negative correlation to conversion rate. We observed a low correlation between conversion rate and the features *new_dale_chall_score* (the New Dale–Chall readability index), *new_dale_chall_class* (the New Dale–Chall readability index interpretation), and *strain_score* (the Strain readability index).
- We found that a low percentage of unique words can cause low conversion, and that short sentences can also cause low conversion. On the other hand, a long text can cause low conversion. A high percentage of difficult words (according to the New Dale–Chall readability index) can also cause low conversion.
- We observed the level of correlation between conversion rate and percentage of unique words to be medium.
- We built a machine learning model using the SVM algorithm. The accuracy of the model was 0.780822, and the *F*_1_ score for each class was 0.783784. The four most important features of the model were *sentences* (number of sentences), *bormuth_score* (the Bormuth readability index), *aws* (average number of words per sentence), and *fog_score* (the Fog readability index). The relationship between conversion rate and the main features of the SVM model is nonlinear.
- We can predict the conversion rate of a landing page by analyzing its text for readability.
- With respect to the report by Unbounce [13], we can only confirm that there is a relationship between the conversion rate and readability of landing page text. There are readability indices that have a positive correlation and readability indices that have a negative correlation to conversion rate. A single readability index can have both a positive and a negative correlation with conversion rate depending on the range of index values.

## 5. Conclusions

This paper showed that text readability analysis can predict conversion rate. A machine learning approach can be applied. A text that is not very readable can result in low conversion. We can increase the conversion rate by increasing or decreasing the readability of the text to adapt it to the target audience’s level of education. Potentially, conversion rate optimization can be done on the basis of the results obtained in this paper. We should recall here that the topic of this paper was not conversion rate optimization, including the conversion rate optimization of landing pages. We were interested in studying the impact of text readability on the conversion rate, and conversion rate prediction.

There are readability indices for English with both positive and negative correlation to conversion rate. Furthermore, a single readability index can have both a positive and a negative impact on the conversion rate. Short sentences and low percentages of unique words can result in low conversion. On the other hand, long text and a high percentage of difficult words (according to the New Dale-Chall readability index) can cause low conversion. The abovementioned conclusions can be useful for increasing the conversion rate.

This paper aimed to investigate whether it is possible to predict the conversion rate by analyzing the readability of a landing page text, rather than presenting a comprehensive review of and detailed instructions on how to improve text readability. The problem of text readability has been widely described in the literature [2,3,38,50].

Five machine learning models were built and evaluated in this paper. The machine learning model based on the use of the SVM algorithm allowed us to classify landing pages according to the predicted conversion with a model accuracy of 0.78, and the *F*_1_ score for each class was 0.78. Additionally, the interpretation of the results of this model was conducted using the SHAP package. This article, among other things, may be suitable for the cold-start problem.

Due to the fact that, in our experiments, at the stage of building machine learning models, only 243 out of 2000 examples were left, the next important step will be to obtain more input data (we hope to have approximately 20,000 examples) in order to confirm the obtained results and to build machine learning models with an accuracy of more than 80%, which is considered sufficient for the production deployment of such a model.

Furthermore, an important step will be the acquisition and analysis of other types of input data containing text and their conversion rate, e.g., e-mails or advertisements. The results may be influenced by obtaining data from only one country, e.g., the United States, due to the existence of different dialects of English. Another direction of development of this study may be the analysis of a broader list of features that can affect the conversion rate, e.g., text type, sentiment, average paragraph length, percentage of stop words, and domain name.

A separate, although promising, direction is to analyze the impact of both text and UI/UX (user interface/experience) design on the conversion rate. Website design has a considerable effect on the immersion a consumer feels and, thus, increases the likelihood of a user staying through conversion [6].

## Figures and Tables

**Table 1:** Example of input data.

Index	Hash	Views	Conversions	CVR
30257	6ccd9b56f798b4e4462c	952	58	0.06092436974789916
28941	8f279a74b7b8b2d7f389	2413	787	0.32615002072109406
12716	2HMbmffc85zQwfbpt4J8	3149	93	0.02953318513813909

**Table 2:** Correlation coefficients between conversion rate and selected variables.

Variable	Pearson Correlation Coefficient	Spearman Correlation Coefficient	Kendall Correlation Coefficient
*new_dale_chall_score*	−0.172655	−0.271096	−0.203832
*new_dale_chall_class*	−0.141998	−0.219499	−0.175616
*strain_score*	0.102520	0.181062	0.136370
*rix_score*	0.088283	0.187938	0.146439
*flesch_score*	0.083050	0.113933	0.077594

**Table 3:** The full list of added features.

Feature	Description	Pearson Correlation Coefficient
*sentences*	Number of sentences	−0.265740
*characters*	Number of characters	−0.195236
*syllables*	Number of syllables	−0.191222
*words*	Number of words	−0.181639
*pdw*	Percentage of difficult words (according to the New Dale–Chall readability index)	−0.176888
*ppw*	Percentage of polysyllabic words	−0.103137
*asw_flesch*	Average number of syllables per word (according to the Flesch readability index)	−0.100058
*pew*	Percentage of echomimetic (onomatopoeic) words	−0.094010
*acw*	Average number of characters per word	−0.085432
*pmw*	Percentage of multicharacter words	−0.068851
*asw*	Average number of syllables per word	−0.055716
*ppw_fog*	Percentage of polysyllabic words (according to the Fog readability index)	−0.029079
*readability_score*	Average readability	−0.018520
*psw*	Percentage of selling words	−0.013047
*asl_flesch*	Average sentence length (according to the Flesch readability index)	0.069256
*ass*	Average number of syllables per sentence	0.094268
*acs*	Average number of characters per sentence	0.094312
*asl_fog*	Average sentence length (according to the Fog readability index)	0.117052
*aws*	Average number of words per sentence	0.117052
*puw*	Percentage of unique words	0.340194

**Table 4:** The quality of the machine learning models.

Model Name	Accuracy	*F*_1_ Score (for Classes 1/0)
Naïve Bayes classifier	0.698630	0.75/0.62
Random decision forest	0.739726	0.77/0.71
Logistic regression	0.739726	0.78/0.69
*k*-NN	0.753425	0.78/0.72
SVM	0.780822	0.78/0.78

## Data Availability

Not Applicable.
